# Public-private partnerships influencing the initiation and duration of clinical trials for neglected tropical diseases

**DOI:** 10.1371/journal.pntd.0011760

**Published:** 2023-11-13

**Authors:** Zhongxuan Ma, Kevin Augustijn, Iwan De Esch, Bart Bossink

**Affiliations:** 1 Breakthrough Tech Innovation research group, Amsterdam Institute of Molecular and Life Sciences, Faculty of Science, Vrije Universiteit Amsterdam, Amsterdam, The Netherlands; 2 Department of Molecular Cell Biology and Immunology, Amsterdam Universitair Medisch Centrum, Amsterdam, The Netherlands; 3 Division of Medicinal Chemistry, Amsterdam Institute of Molecular and Life Sciences, Faculty of Science, Vrije Universiteit Amsterdam, Amsterdam, The Netherlands; George Washington University School of Medicine and Health Sciences, UNITED STATES

## Abstract

Public-private partnerships (PPPs) for neglected tropical diseases (NTDs) are often studied as an organizational form that facilitates the management and control of the huge costs of drug research and development. Especially the later stages of drug development, including clinical trials, become very expensive. This present study investigates whether and how the type of PPPs influences the initiation and duration of NTD clinical trials. Using the ClinicalTrials.gov database, a dataset of 1175 NTD clinical studies that started between 2000 and 2021 is analyzed based on affiliation information and project duration. For the NTD clinical trials that resulted from PPPs, the collaborating types were determined and analyzed, including the public sector-, private sector-, governmental sector-, and nongovernmental organization-led collaborations. The determinants for the discontinuation of all stopped clinical trials were categorized into scientific-, funding-, political-, and logistic dimensions. The results reveal that public sector-led PPPs were the most common collaborative types, and logistic and scientific issues were the most frequent determinants of stopped clinical trials.

Trial registration: ClinicalTrials.gov.

## Author summary

In the present study, we focused on understanding the development of collaborative clinical trials for neglected tropical diseases that affect hundreds of millions of people each year. These diseases, largely prevalent in tropical regions, lead to huge economic and societal burdens. The current status of drug development in this field is caused and exacerbated by a combination of diverse political, market, and scientific factors. Our goal was to highlight the crucial role of collaborations between public entities and private organizations in initiating and promoting clinical trials for these neglected diseases. Our results show that public sector and nongovernmental organization-led collaborations were the most prevalent between 2000 and 2021. Logistic and scientific issues are the most frequent reasons for failures in this clinical trial dataset. In conclusion, our study paints an overview picture of the collaborative clinical trials against neglected tropical diseases. The results highlight the power of collaboration and innovation in addressing this kind of health need. We aspire that our insights will spark more conversations and initiatives aimed at providing equal healthcare opportunities for everyone.

## 1 Introduction

Neglected tropical diseases (NTDs) are defined as a group of viral, bacterial, and parasitic infection diseases across low and middle-income countries, which have affected hundreds of millions of people worldwide and present a substantial economic burden each year [1–3]. They significantly impact marginalized groups, exacerbate their difficulties [4], and impose a significant disease burden, often rivaling that of major global health concerns such as *AIDS*, *Tuberculosis*, and *Malaria*, especially within low-income countries where they predominantly affect rural and urban regions [5]. NTDs not only impede human development but have historically been under-researched, despite their profound impact [6]. Given this, addressing NTDs is of paramount importance, not solely from a health perspective, but also from a treatment perspective, underscoring the pressing need for further development of medical interventions and R&D practices [4,7].

The status of the NTD-related drug development industry is influenced by a combination of diverse political, market, and scientific factors. A notable proportion of global health funding is nowadays allocated to *AIDS*, *Tuberculosis*, and *Malaria* that (may) occur in low, middle, and high-income countries and show more favorable return-on-investment opportunities [8]. Yet, NTDs, which also have a broad impact, but mainly in low-income countries, are apportioned a comparatively low share of funding. The low return on investment of NTD medicines for low-income countries and insufficient knowledge of the diseases are among the factors that discourage the pharmaceutical industry from investing in related research and development (R&D) programs [9–12]. The considerable R&D financial commitments that are requested, along with the often less-than-favorable evaluations of Net Present Value (NPV), further accentuate the cautious approach toward public and private investments [9,10]. Market dynamics are also active. The limited commercial prospects and the relative scarcity of incentives might not sufficiently allure R&D commitments from potential funders and developers [11,12].

Considering these situational factors, the public-private partnership (PPP) is progressively recognized as an effective organizational form that enables stakeholders to bridge existing gaps in NTD drug development by collaboratively aligning public health goals and private commercial interests [12–14]. The PPP form of collaboration became more and more common around the early 2000s with for example the advent of product development partnerships like the *Medicines for Malaria Venture* (MMV) [13,14]. Such PPPs amalgamated the efforts of charities, industry, and academic groups, focusing on specific communicable diseases that predominantly affect less affluent nations [15,16]. Facilitated by PPPs, the synergy between these stakeholders, underpinned by rational criteria for lead progression, became central in advancing drug development for NTDs [16–18].

The regularly recurring argument is that public-private partnerships (PPPs), defined as collaborative legally binding agreements between diverse governmental and commercial organizations, play an important role in addressing NTD issues over the past few decades [15–21], and foster the development and accessibility of innovative, affordable, and user-friendly medical solutions for NTDs by sharing and distributing resources, knowledge, risks, and benefits [22]. The stakeholders in these PPPs encompass international research centers, governmental agencies, non-governmental organizations (NGOs), pharmaceutical firms, and academic institutions [16]. Some PPPs, such as for instance the *Dutch Top Institute Pharma*, have been leading in promoting the NTD R&D pipeline by establishing a multi-stakeholder platform where participants are enabled to pool resources and synergize their efforts [14]. Other PPPs, for example, have combined the strengths of NGOs and governments to enhance the accessibility of essential medicines by offering necessary training and services [23]. However, the transparency of these PPPs for NTD-related R&D initiatives often remains limited, which poses challenges, and is a starting point for comprehensive empirical studies in this domain [24,25].

As touched on, the prominence of PPPs can be traced back to the transitional period of the late 1990s and early 2000s, when several PPP-models for neglected tropical diseases were emerging [26–28]. This evolution was facilitated by various governmental and commercial organizations that participated in public-private partnerships [28], and the emergence of PPPs during this era offered collaborative platforms that enabled the sharing and distribution of resources and expertise from diverse sectors, including academia, international organizations, and the private industry [29]. Although these partnerships facilitated access to treatment and were instrumental in the global search for solutions to NTDs, these were also not without criticism. For example, it is reported that while PPPs made strides in solving NTD problems, their success is strongly contingent upon a policy environment that fosters collaboration and engagement from all stakeholders [30]. And potential drawbacks may occur if partners mainly participate out of self-interest; do not take responsibility and instead place that responsibility with other partners; use the PPP as a form of Corporate Social Responsibility (CSR) window dressing; aim to develop specialist knowledge and shield it from competitors; or, are solely interested in accessing additional funding through the PPP [31,32]. As the global health community continues to leverage the advantages of PPPs for NTDs, it would be important to critically evaluate these PPPs impact, benefits, and potential drawbacks to ensure that they truly serve the populations suffering from NTDs [29].

Considering the World Health Organization’s *Neglected Tropical Diseases Roadmap for 2021–2030*, it becomes imperative to delve deeper into the strategies proposed for enhancing access to safe, effective, and quality-assured medical products [33]. The emphasis on medical products is rooted in the understanding that timely and effective interventions can substantially mitigate the health burdens of NTDs. PPPs emerge as a core mechanism in this roadmap, given their potential to foster collaborative efforts, pool resources, and expedite the development and delivery of essential medical products for NTDs [15,18]. Although more and more participating stakeholders are making contributions, collaborative models and governance systems are still loosely structured [22,25]. Due to the growing importance of PPPs and the relatively limited available knowledge about how to control and increase their effectiveness, identifying the influential cooperation models and determinants from an evidence-based perspective can help policy-makers and practitioners to develop suitable NTD-related R&D policies and strategies [15,16,20].

NTD issues often transcend national boundaries [23,34], and within this context, the emergence and role of PPPs have been growing [13,18,21]. Understanding the dynamics and implications of these PPPs is not only crucial for neglected tropical diseases but also offers insights into the broader landscape of global health governance and policy [13,15]. This study, by delving into the determinants of PPPs for NTD-related clinical trials, aims to offer insights. By shedding light on the collaborative types of these PPPs, this present study provides suggestions for PPP stakeholders to initiate collaborative clinical trials, optimize resource allocation, and ultimately, foster improved NTD health-related outcomes for vulnerable areas. Deeper insight into the public-private partnership dynamics can serve as a reference for strategizing NTD-related public health interventions, aiming to catalyze meaningful advancements in global health [15,18,35].

This study uses well-documented clinical trials to investigate if the type of PPPs influences the initiation and duration of NTD clinical studies. By merging and analyzing NTD clinical trial data, it extracts insights and draws lessons for future research.

## 2 Methods

### 2.1 Data collection, processing, and analysis

In line with an evidence-based perspective, the present study builds a dataset of clinical studies from *ClinicalTrials*.*gov* of the *National Institutes of Health (NIH)*. First, we adopted the *United Nation’s World Health Organization* (WHO) classification, which divides NTDs into twenty categories (see S1 File). For each disease category, we built a list of search terms that appear in the research literature, patents, and clinical reports. Different abbreviated forms of pathogen names and disease names are also considered to find as many clinical trials as possible. For example, the search terms for *Trypanosoma* include ‘*Brucei*’, ‘*Chagas disease*’, ‘*Chagasi disease*’, ‘*Chagas disease*’, ‘*Cruzi*’, ‘*Gambiense*’, ‘*Kissing bugs*’, ‘*Rhodesiense*’, ‘*Sleeping sickness*’, ‘*Triatominae*’, ‘*Tripanosomiasis*’, ‘*Trypanocidal*’, ‘*Trypanocides*’, ‘*Trypanosoma*’, ‘*Trypanosomatid*’, ‘*Trypanosome*’, ‘*Trypanosomiasis*’, ‘*Trypsonosomia*’, ‘*Tsetse fly*’, and ‘*Winterbottom*’. In this way, a table containing twenty disease-related keywords was established (see S1 Table), which built an initial dataset that contains 3972 clinical trials. Then the dataset was scanned manually and both the missing and overlapping trials were removed. The clinical trials that lack ‘locations’ or ‘information provider’ or ‘sponsors/contributors’ were filtered out from the dataset, because these clinical trials do not fit the purpose of this study. It is pertinent to note that during the data filtration process, we did not consider the type of product involved in each trial. The dataset was collected in February 2022 and the final dataset contains 1175 clinical studies that started between 1/1/2000 and 31/12/2021.

To study whether the type of PPPs influences the initiation and duration of NTD clinical studies, the 1175 NTD-related clinical trials were analyzed concerning affiliation information and project duration. According to previous literature, information limitations of PPP-datasets make it difficult to directly list all the partners within PPP-initiated projects [25], and thus the present study identifies the partners based on the data of ‘sponsors/contributors’ and ‘responsible party’. To illustrate, the data of ‘sponsors/contributors’ indicates the partnering contributors towards each clinical study, while ‘responsible party’ indicates the organizations that are responsible for the clinical project. If the affiliation names of ‘sponsors/contributors’ and ‘responsible party’ are the same type of organizations, the clinical trial is classified as ‘Non-PPP’. If the affiliation names of ‘sponsors/contributors’ and ‘responsible party’ are of different types of organization, the clinical trial is classified as ‘PPP’.

For the PPP sub-dataset, based on the collection of ‘responsible party’ and ‘sponsors/contributors’, the collaborating types were determined and analyzed, including the public sector-, private sector-, governmental sector-, and NGO-led collaborations. For example, the responsible party of NTD project *NCT00146627* is the public organization ‘*Drugs for Neglected Diseases’* and the collaboration includes six public and private organizations. Therefore, this clinical study is classified as a public sector-led collaboration. The present study then separated ‘stopped’ studies and categorized the determinants based on the reasons shown in the section ‘recruitment status’ of the dataset. The present study extracted these words and then reviewed all the cited references in the clinical dataset to determine if these references support the reasons for discontinuation. The determinants were classified into four dimensions, which are ‘scientific’, ‘funding’, ‘political’, and ‘logistic’ (see S2 Table). For instance, the terminated reason of NTD project *NCT01539161*, ‘*extremely low enrollment rate*’, is classified as ‘logistic’.

### 2.2 Ethics statement

In the Netherlands, ethics review is only required by the Medical Research Involving Human Subjects Act (WMO) for (medical) research involving humans or animal models. Because this study is non-medical, it is exempt from ethics review. The research methodology of the present study is based on a public online dataset, including research design, data collection, and data coding. In addition, this study does not involve vulnerable groups or confidential data.

## 3 Results and discussion

### 3.1 NTD-related PPP subtypes

**Fig 1:**
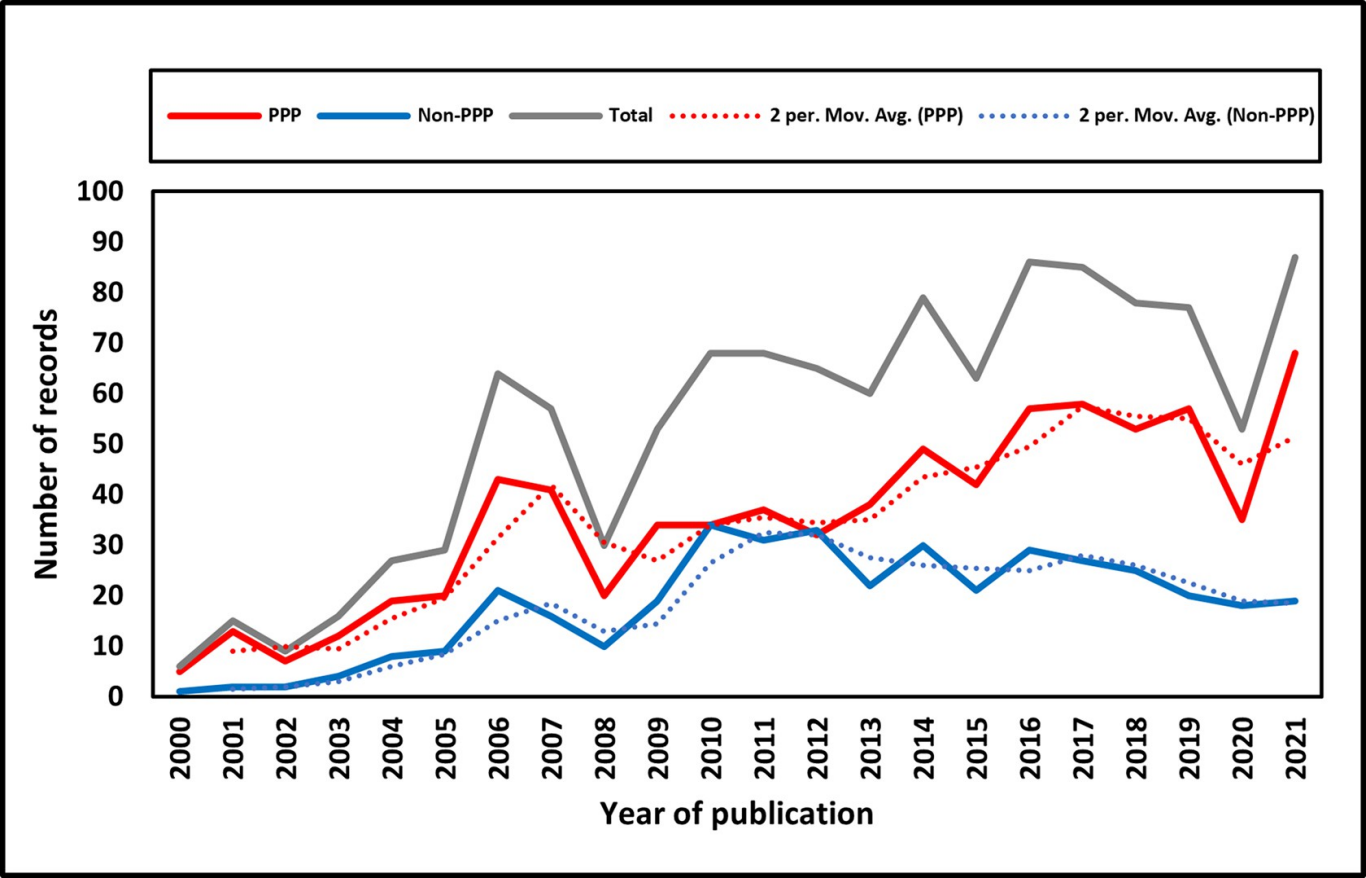
Newly initiated NTD-related clinical trials per year. The total annual number is shown as the grey solid line, the number of PPP programs (n = 774) as the red solid line, the two-period moving average trendline as the red dotted line, the number of Non-PPP programs (n = 401) as the solid blue line, and the two-period moving average trendline as the blue dotted line. The Y-axis describes the number of clinical trials, and the X-axis indicates the newly initiated year.

The number of NTD-related Non-PPP (n = 401) and PPP-initiated (n = 774) clinical studies are shown in Fig 1. As is shown by the dotted trendlines in Fig 1, the number of NTD-related Non-PPP and PPP-initiated clinical studies show a similar degree of increase before 2010. From 2011 onwards, the number of PPP-initiated clinical studies continued to rise, while the number of Non-PPP-initiated clinical trials appeared to decline. Despite a dip in PPPs around 2020 at the time of the Covid-19 pandemic, the number of PPPs has subsequently risen steadily again.

Compared with traditional or single-institute-initiated clinical trials, PPPs have several advantages. For example, a study by Aerts et al. proposes that PPPs have the capacity to leverage each participant’s comparative advantage(s), resulting in more efficient and effective product development and distribution for NTDs [25]. Furthermore, PPPs encourage investment in both R&D as well as product delivery. Within PPP-initiated collaborations, the synergy between the R&D and delivery processes is essential for the elimination of NTDs [25]. Until 2019, the total number of newly initiated clinical trials has been fluctuating at the plateau level, with the annual number exceeding sixty.

**Fig 2:**
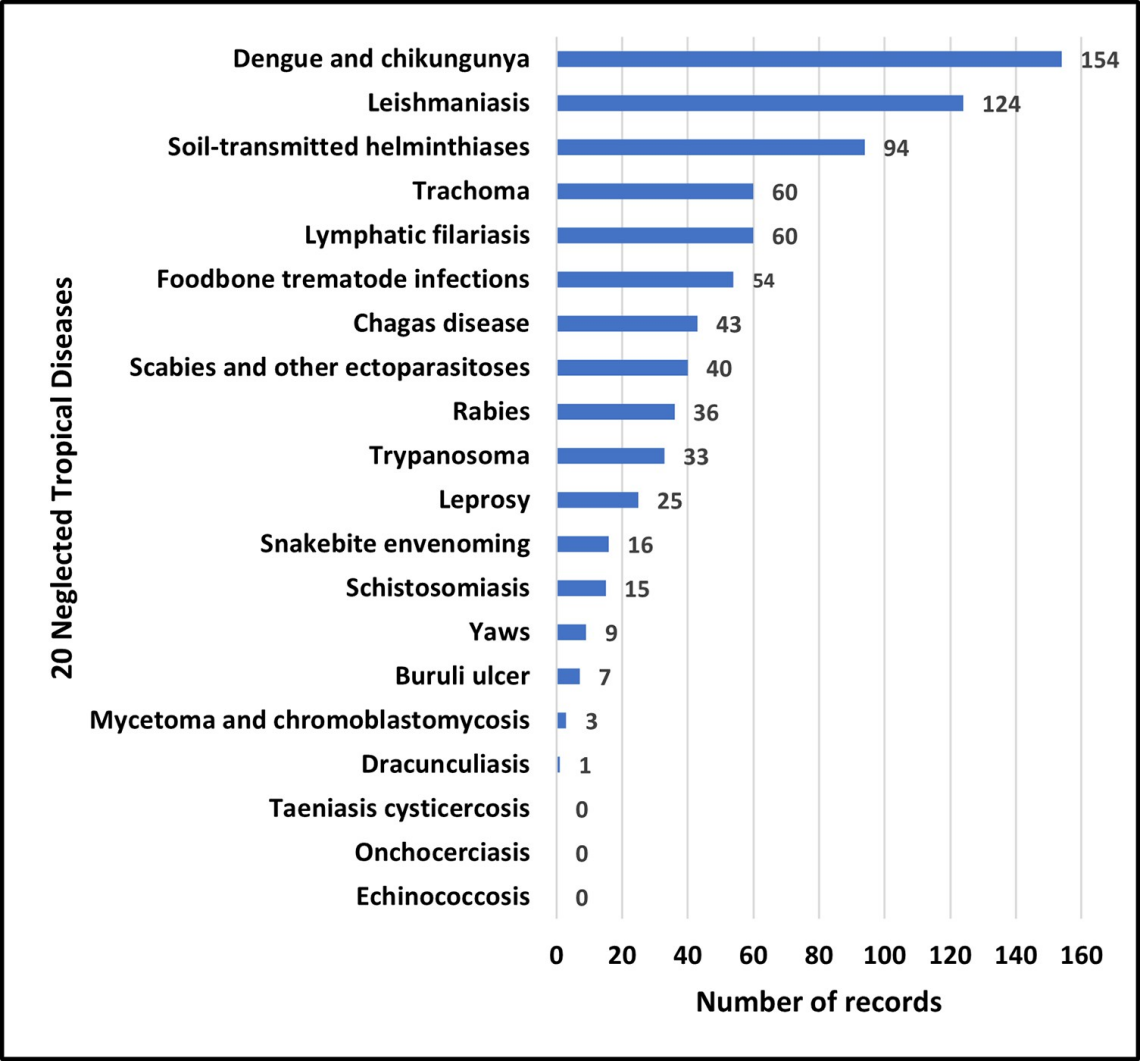
Number of PPP-initiated NTD clinical trials from 2000–2021. The 20 NTDs are listed on the Y-axis in descending order. The number of PPP-initiated clinical trials for each disease is shown separately next to each bar.

As is shown in Fig 2, it is observed that diseases such as *Dengue* and *Chikungunya* have garnered a relatively high number of PPPs, amounting to 154 clinical studies between 2000 and 2021. Similarly, the number of PPP-initiated clinical trials for *Leishmaniasis* is 124 (Fig 2). This could potentially indicate a prioritization of these diseases in the realm of public-private partnerships. On the other hand, certain diseases, including *Taeniasis cysticercosis*, *Onchocerciasis*, and *Echinococcosis*, have not been associated with PPP-initiated clinical trials during this timeframe, indicating areas where further research and partnership efforts could be beneficial. It is noted that Fig 2 only displays the number of clinical studies for the 20 PPP-initiated NTDs and does not show the percentage of PPPs for each disease out of the total clinical studies of each disease.

**Fig 3:**
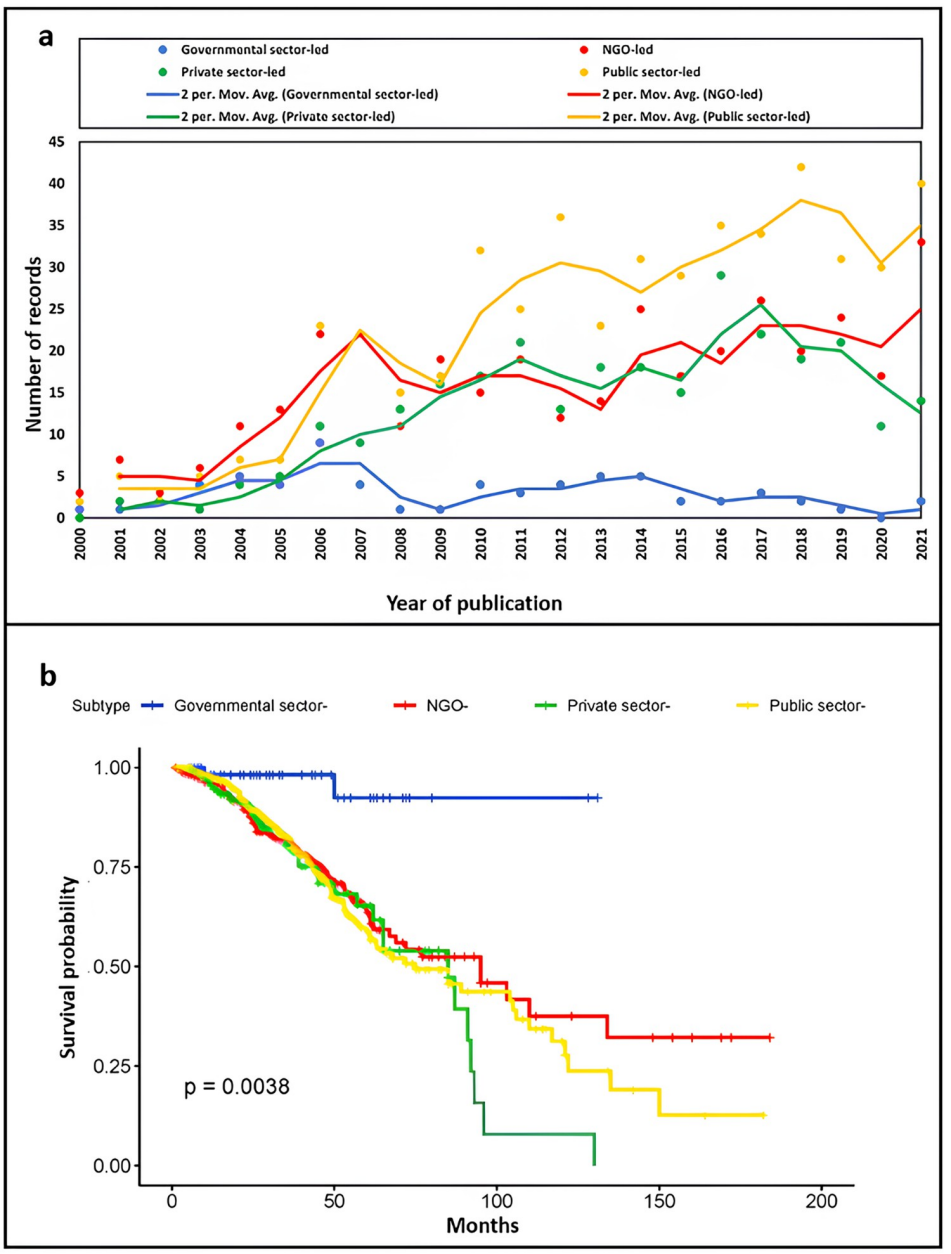
Development of NTD-related clinical trials established by PPP subtypes in the dataset. **(a)** The clinical dataset is separated into the governmental sector- (blue dots), NGO- (red dots), private sector- (green dots), and public sector- (yellow dots) led collaborations. The corresponding-colored trend lines are calculated by the average of the trials every two consecutive years. **(b)** The duration of clinical trials of different collaborative types, analyzed by survival between the starting and completion dates.

As is shown in Fig 3A, the mainstream of NTD-related collaborative studies consists of collaborations led by public sector organizations and NGOs. As is shown in Fig 3B, the public sector-led collaborations tend to terminate later, and private sector-led collaborative projects have a shorter duration.

With rising investments coming from both public and private stakeholders, PPPs are expected to continue to show their promise in the coming years [36–39]. In private or public sector-led collaborations, academic and industrial scientists are more likely to work together for NTD-related collaborative studies under an agreement [16]. The collaboration between organizations in public and private sectors provides a productive environment, integrating scientific research with public demand in NTDs, which supports NTD-related novel therapeutics and patents [16].

According to the previous literature, NGOs and governmental sector organizations support researchers to develop innovative and unproven approaches with support from governmental organizations, local communities, and philanthropic organizations [22,26,40,41]. Most NGOs act as the coordinating entity, providing funding and platform support, with some also employing researchers. A promising feature of governmental sector-led collaborations is that they can emulate the R&D programs of biopharmaceutical companies and leverage existing capabilities in disease-endemic countries [26]. Take one cross-boundary initiative (Uniting to Combat NTDs consortium) as an example, the involvement of governments aims to facilitate the delivery of donations to the populations in need, though its effectiveness can vary [27].

### 3.2 Determinants of clinical trial discontinuation of NTD PPP collaborations

**Fig 4:**
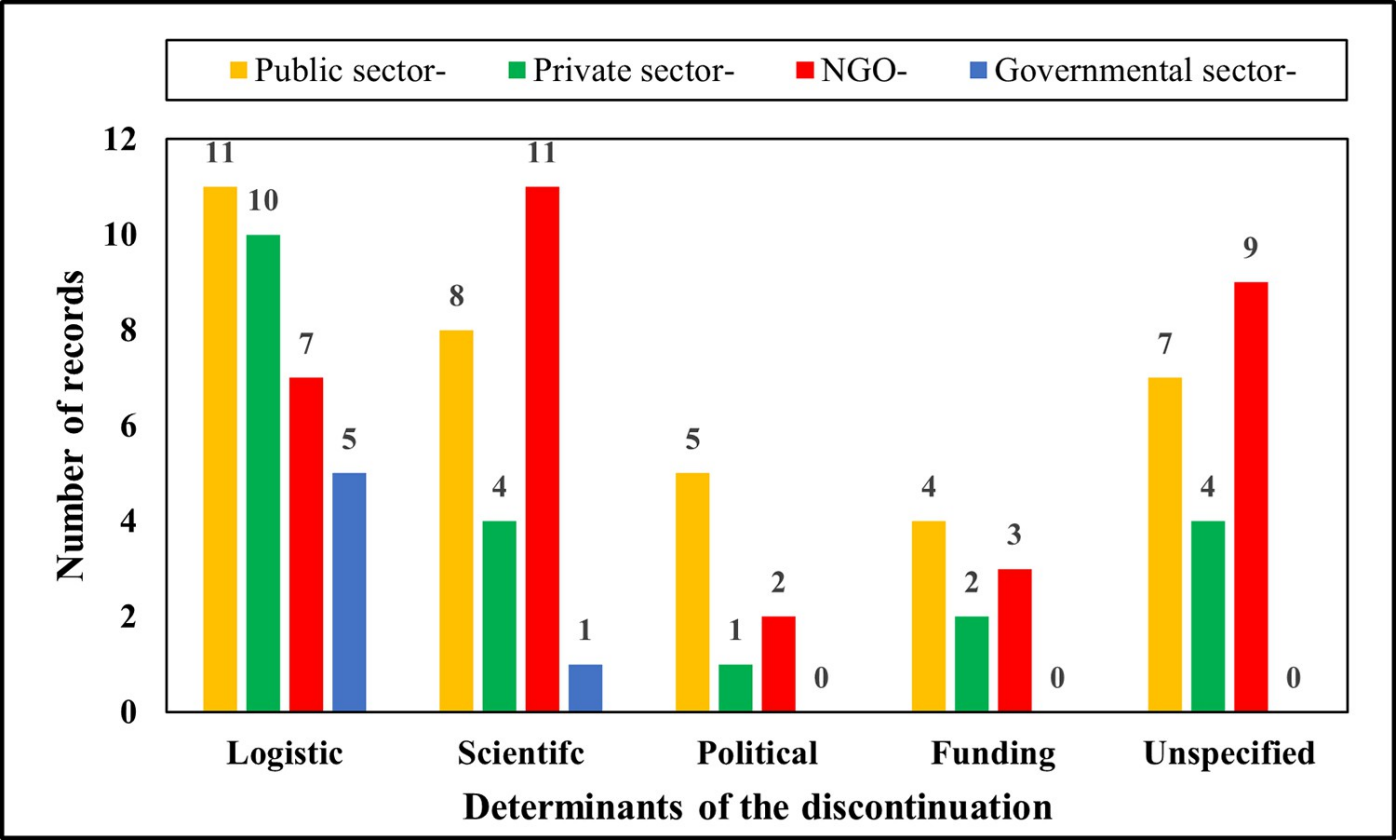
Influential dimensions identified from the dataset. The vertical coordinate indicates the number of records of newly initiated PPP NTD-related clinical trials. The horizontal coordinate indicates five dimensions that influence the progress of PPP NTD-related clinical trials are identified by the dataset, including logistic- (n = 33), political- (n = 8), funding- (n = 9), scientific- (n = 24), and unspecified- (n = 20) dimensions.

Within the dataset, some of the stopped clinical trials do not specify the reasons for discontinuation, while others mention these reasons with a short phrase or sentence. This study extracted 94 discontinued clinical trials, which mentioned the reason for discontinuation. The other 680 trials were either completed or ongoing at the time of analysis. The present study extracted the words from these reasons written in ‘Recruitment Status’ and summarized determinants of the discontinuation of stopped NTD-related clinical trials into logistic-, political-, funding-, and scientific- dimensions (Fig 4).

#### 3.2.1 Scientific dimensions

Within the dataset, ‘scientific determinants’ refer to the factors related to the efficacy, safety, and scientific decision-making of a clinical trial, including the most frequent words ‘*low efficacy*’, ‘*safety*’, and ‘*disease*’ (Table 1). These factors can have an impact on the progress of NTD-related clinical trials by influencing the development of effective and safe treatments. Understandably, NTD-related clinical trials are performed and advanced in the form of a series of experiments. When considered as influential factors, scientific dimensions are significantly important. For instance, drug safety affects the progress of clinical trials because serious adverse events or side effects can lead to delays or even discontinuation of the clinical trials [42]. Low efficacy rates can halt the progress of a clinical trial by making it difficult to demonstrate statistically significant differences between the treatment and control groups [43]. To optimize the progress of clinical trials, it is important to prioritize both drug safety and efficacy, and to work closely with regulatory agencies and clinical trial participants to ensure that trials are conducted efficiently and with patient safety as the top priority.

**Table 1:** A summary of determinants.

Dimensions	Top-used words	Public-	private-	Nongovernmental-	Governmental-	Number
**Scientific-**	Low efficacy, Safety, Diseases.	8	4	11	1	24
**Political-**	Regulatory, Administration.	5	1	2	0	8
**Funding-**	Funding, Accrual, Sponsor.	4	2	3	0	9
**Logistic-**	Enrollment, Covid-19, Recruitment.	11	10	7	5	33
**Unspecified-**		7	4	9	0	20

#### 3.2.2 Political dimensions

As is shown in Table 1, ‘political determinants’ refer to external factors related to regional regulations (‘*regulatory*’) and local administration (‘*administration*’) that may delay and impede the progress of clinical trials for NTDs, especially in regions with insufficient health infrastructure or travel restrictions. The role of relevant regulations and incentives should not be neglected because NTD-related global commitments, such as *WHO Roadmap Goals* and *Global Plan to Combat Neglected Tropical Diseases 2008–2015*, help to repair market failure and provide diverse collaborative models for NTD clinical studies [3,41]. Since the beginning of the twenty-first century, various forms of PPPs have been the major contributors to NTD-related R&D. Besides initiating multi-stakeholder PPPs, this present study suggests that the investment in vaccinations and diagnostics in low and middle-income countries is effective as well [3,22], towards which governments can align their organizing and regulating contributions.

#### 3.2.3 Funding dimensions

‘Funding dimensions’ refer to influential factors related to financial support that led to the stop, such as the most frequent words ‘*funding*’, ‘*accrual*’, and ‘*sponsor*’ (see Table 1 and Fig 4). These determinants impact the ability of researchers to conduct and promote the trials as planned. Especially for NTD-related clinical trials, the adequacy and sustainability of the funding are considered to be among the most important determinants [18,44]. Current foundations and political incentives have successfully mobilized global efforts to handle neglected tropical diseases in terms of intervention, R&D, and governance [17]. Within the dataset, some NTD-related clinical programs are suspended or terminated due to the lack of ongoing funding. This is because, among others, it is difficult for policymakers to evaluate which projects are more ‘fundable’ [45]. Without funding support, it would be difficult for any collaborative consortium to obtain a good R&D performance [17,46]. Therefore, at the policy level, it is relevant to allocate more financial support towards NTDs; while at the practice level, practitioners may pay more attention to optimizing their R&D strategy to reduce costs, such as developing or repurposing promising therapies already in their pipelines [22].

#### 3.2.4 Logistic dimensions

Some determinants can be classified in the cluster titled ‘logistic dimensions. The most frequent words are ‘*enrollment*’, ‘*covid-19’*, and ‘*recruitment*’ (Table 1). By classifying these influential factors as ‘logistic dimensions’, the present study groups together determinants that are related to practical and managerial issues. Toor and co-authors predicted that during the Covid-19 period ongoing and planned PPP NTD trials could be confronted with challenges in enrolment, funding, and execution [36–38]. The logistic determinants arise during the clinical trial, which may impact the conduct and outcome of the trial. There are several common and important issues when managing PPPs in such a typical healthcare system [47,48]. These include selecting and reviewing the projects, negotiating partnership agreements, managing the interfaces of drug R&D, allocating project portfolios, and integrating upstream and downstream interfaces [49]. According to the previous literature, managers, and coordinators may focus on developing organizational dynamic capabilities in R&D collaborative project portfolio management, which promotes NTD clinical trials and drug availability [22,50–52].

### 3.3 Contribution and limitation of this study and suggestions for future research

While there exists some literature on the dynamics of PPPs in health research, to the best of our knowledge, this research is among the first to offer an in-depth analysis specifically tailored to NTDs. A distinctive feature of this study is its dual focus: it examines the collaborative types of the initiation of NTD clinical trials and the determinants based on discontinued clinical trials. The findings presented aim to contribute to the academic dialogue and also provide suggestions for stakeholders in the NTD research arena, facilitating the effective initiation and continuation of such collaborations. Despite the observed increase in PPP R&D activities related to NTDs, the present study suggests policymakers to release more adequate incentives, encompassing both push and pull strategies like *Priority Review Vouchers* to attract R&D investments from funders and product developers [53].

Next to its merits this study also has its limitations and based on that several research suggestions can be made. First, the present study uses a keyword-based searching strategy to collect NTD-related clinical trials, which may result in missing relevant trials that do not include the chosen keywords. Moreover, the study only includes clinical trials with available sponsors, contributors, and information providers, which might lead to selection bias when a clinical study does not provide the information therein. Future research is suggested to enrich the dataset by including these clinical trials. Secondly, the method deployed by the present study does not extract and analyze all PPP-initiated studies. For example, if a PPP develops a drug candidate and only one partner brings it into clinical trials, this study’s methodology will not classify it as a ‘PPP-initiated clinical trial’. Therefore, future research can further optimize data classification methods. Thirdly, this study makes academic recommendations for the initiation of collaborative R&D in PPPs. However, it only uses NTDs as the case situation and the results may not be generalizable to clinical trials in other disease areas. Therefore, it is important for future research to fill this gap. Finally, the dimensions explored in this study were derived from clinical trial databases, which adhere to stringent standards and conventions recognized by professionals and regulatory authorities. Incorporating additional qualitative data sources in future research, such as semi-structured interviews and archival data, could additionally provide insights into factors that might not be prominently documented in formalized written repositories.

## 4 Conclusions

Based on a dataset related to clinical trials, the present study analyzes the NTD-related R&D PPPs concerning the research question: does the type of PPP influence the initiation and duration of NTD clinical trials? The results suggest that public sector-led PPPs were the most prevalent, followed by NGO-led PPPs, and private sector-led PPPs. Private sector-led PPPs seem to be more likely to terminate their trials earlier than publicly-led PPPs, towards which future in-depth research is needed. Logistic and scientific issues are the most frequent determinants of stopped clinical trials, followed by funding and political issues. The study contributes to the existing literature by providing insights into the impact of PPP types on NTD-related R&D practices and highlights the significance of addressing logistic issues in clinical trials. These insights contribute to academic, managerial, and political practitioners with an understanding of initiating NTD-related clinical trials and providing future research directions. For academia, the present study suggests that integration and communication between experts from different fields will improve the efficiency of NTD-related clinical trials. Policymakers can notice the role of pharmaceutical companies and initiate relevant incentives for industry-based R&D collaborations. Pharmaceutical companies have acted as stakeholders in this research field for a long time, either working with other public-private partnerships or within their boundaries. However, few publications focus on how pharmaceutical companies manage NTD-related R&D PPPs, which future empirical studies can find out.

## Supporting information

S1 Table Searching terms for twenty neglected tropical diseases. (DOCX)

S2 Table Examples of determinants of different dimensions. (DOCX)

S1 File Dataset. (XLSX)
