# Supplementary material for: Public-private partnerships influencing the initiation and duration of clinical trials for neglected tropical diseases
Source: PLoS Negl Trop Dis. 2023 Nov 13;17(11):e0011760. doi: 10.1371/journal.pntd.0011760 (PMC10681307; doi:10.1371/journal.pntd.0011760)
Supplement: S1 Table — (DOCX) [file pntd.0011760.s001.docx]

**S1 Searching terms for twenty neglected tropical diseases^1^**

| **No.** | | **Disease name** | | **Searching Terms** | |
| --- | --- | --- | --- | --- | --- |
| 1 | | ***Buruli ulcer*** | | Buruli ulcer, Bairnsdale ulcer, Daintree ulcer, Mossman ulcer, Kumasi ulcer, Searls ulcer, Mycobacterium ulcerans, M. ulcerans, mycolactone, Haemophilus ducreyi. | |
| 2 | | ***Dengue and chikungunya*** | | Dengue, Severe dengue, breakbone fever, vomiting and dandy fever, dengue hemorrhagic fever, dengue shock syndrome, chikungunya, CHIKV, Chikungunya virus. | |
| 3 | | ***Dracunculiasis*** | | Dracunculiasis, Guinea-worm disease, Guinea worm, Dracunculus medinensis. | |
| 4 | | ***Echinococcosis*** | | Echinococcosis, cystic echinococcosis, unilocular echinococcosis, Echinococcus granulosus sensu lato, alveolar echinococcosis, alveolar colloid of the liver, alveolar hydatid disease, alveolococcosis, multilocular echinococcosis, small fox tapeworm, Echinococcus multilocularis, polycystic echinococcosis, human polycystic hydatid disease, neotropical echinococcosis, Echinococcus vogeli, Echinococcus oligarthrus. | |
| 5 | | ***Foodbone trematode infections*** | | Trematodiases, Buski, Echinostoma, Metagonimus, Heterophyes, Gastrodiscoides, Lung flukes, Clonorchiasis, Opisthorchiasis, Fascioliasis, Paragonimiasis, intestinal flukes, lung flukes, liver flukes, Clonorchis, Opisthorchis, Fasciola, F. buski, Echinostoma, Metagonimus, Heterophyes, Gastrodiscoides. | |
| 6 | | ***Trypanosoma*** | | Trypanosoma brucei, human African trypanosomiasis, Sleeping sickness, Brucei, Gambiense, Tsetse fly, Winterbottom, Rhodesiense. | |
| 7 | | ***Chagas disease*** | | Chagasi disease, Chagas disease, Tripanosomiasis, Trypanocidal, Trypanocides, Trypanosoma cruzi, Trypanosomatid, Trypanosome, Trypanosomiasis, Trypsonosomia, Triatominae, Kissing bugs, Cruzi. | |
| 8 | | ***Leishmaniasis*** | | Aleppo Boil, Aleppo Button, Aleppo Evil, Aleppo ulcer, Antileishmanial, Bay sore, Chiclero ulcer, Chiclero’s ulcer, Dicera de Baurid, Donovani, Forest yaws, Infantum, Kala azar, Kala-azar, Leishmania, Leishmanials, Leishmaniases, Leishmaniasis, Leishmanicidal, Leishmanicide, Leishmaniosis, Leismaniasis, Oriental sore, Pian bois, PKDL, Tropical sore. | |
| 9 | | ***Leprosy*** | | Leprosy, Hansen's disease, paucibacillary, multibacillary, indeterminate leprosy, tuberculoid leprosy, borderline tuberculoid leprosy, borderline leprosy, Mid-borderline leprosy, borderline lepromatous leprosy, lepromatous leprosy, Mycobacterium leprae, Mycobacterium lepromatosis. | |
| 10 | | ***Lymphatic filariasis*** | | Lymphatic filariasis, Elephantiasis tropica, elephantiasis arabum, filarial worm, Wuchereria bancrofti, Brugia malayi, Brugia timori. | |
| 11 | | ***Mycetoma, chromoblastomycosis and other deep mycoses*** | | Mycetoma, mycetoma belt, eumycetoma, Madurella mycetomatis, Nocardia brasiliensis, Actinomadure madura Streptomyces somaliensis, Actinomadura pelletieri, Chromoblastomycosis, chromomycosis, Carrión mycosis, Lane–Pedroso mycosis, verrucoid dermatitis, black blastomycosis, F pedrosoi, C carrionii, P verrucose, deep mycoses. | |
| 12 | | ***Onchocerciasis*** | | Onchocerciasis, River blindness, Robles disease, Onchocerca volvulus. | |
| 13 | | ***Rabies*** | | Rabies, Rabies lyssavirus, hydrophobia, rabhas, rabere, neurotropic virus, Lyssa Virus, lyssa. | |
| 14 | | ***Scabies and other ectoparasitoses*** | | Scabies, Human scabies, Sarcoptes scabiei, Norwegian scabies, common scabies, Ectoparasites, Cimex lectularius, fleas, lice. | |
| 15 | | ***Schistosomiasis*** | | Bilharzia, Bilharziose, Blood fluke, Blood flukes, Cercaria, Haematobium, Intercalatum, Katayama disease, Mansoni, Mekongi, S. bovis, S. Japonicum, Schistosoma, Schistosomal, Schistosome, Schistosomiasis, Schistosomula, Schistosomulum, Snail fever, Trematoda. | |
| 16 | | ***Soil-transmitted helminthiases*** | | worm infection, helminthiases, Hookworm, Ascaris, whipworm, Ascaris, Ascaris lumbricoides, Soil-transmitted helminthiases, lymphatic filariasis, dracunculiasis, onchocerciasis, nematodiasis, cestodiasis, trematodiasis, schistosomiasis, fascioliasis, clonorchiasis, opisthorchiasis, paragonimiasis, cysticercosis, taeniasis, echinococcosis, Acanthocephala infection, Trematode infection, food-borne trematodiases, Tapeworm infection. | |
| 17 | | ***Snakebite envenoming*** | | Snakebite envenoming, snake venoms, hemotoxic, neurotoxic. | |
| 18 | | ***Taeniasis cysticercosis*** | | Cysticercosis, Taeniasis cysticercosis, taeniasis, tapeworm infection, cestode, Taenia solium, T. saginata, Cysticercus bovis, T. ovis, Cysticercus ovis. | |
| 19 | | ***Trachoma*** | | Trachoma, Granular conjunctivitis, blinding trachoma, Egyptian ophthalmia, Chlamydia trachomatis. | |
| 20 | | ***Yaws*** | | Frambesia tropica, Yaws, Endemic Treponematoses, thymosis, polypapilloma tropicum, non-venereal endemic syphilis, parangi, paru, bouba, frambösie, pian, frambesia, bakataw, Treponema pallidum pertenue. | |

**Reference:**

1. Related links: Neglected Tropical Diseases [Internet]. World Health Organization [cited 2023 Nov 1]. Available from: <https://www.who.int/teams/control-of-neglected-tropical-diseases>
