# Supplementary material for: Public-private partnerships influencing the initiation and duration of clinical trials for neglected tropical diseases
Source: PLoS Negl Trop Dis. 2023 Nov 13;17(11):e0011760. doi: 10.1371/journal.pntd.0011760 (PMC10681307; doi:10.1371/journal.pntd.0011760)
Supplement: S2 Table — (DOCX) [file pntd.0011760.s002.docx]

**S2 Examples of determinants of different dimensions**

| **Dimension name** | **Determinant example** |
| --- | --- |
| Scientific dimension | *“safety concerns”, “low effective”, “lack of efficacy”,*  *“Termination of the diseases”, “low cure rate”, “poor tolerability”, etc.* |
| Political dimension | *“local security situation”, “local regulatory”, “local administration”,*  *“lack of authority”, “governmental decision”, “political issues”, etc.* |
| Funding dimension | *“funding stop”, “funding stop”, “insufficient accrual”,*  *“lack of VC funding”, “insufficient accrual”, “sponsor decision”, etc.* |
| Logistic dimension | *“lack of recruitment”, “supply constraints”, “COVID-19”,*  *“lockdown”, “low enrollment rate”, “lack of supply”, etc.* |
